# A Rainwater Harvesting and Treatment System for Domestic Use and Human Consumption in Native Communities in Amazonas (NW Peru): Technical and Economic Validation

**DOI:** 10.1155/2021/4136379

**Published:** 2021-10-19

**Authors:** Eli Morales Rojas, Edwin Adolfo Díaz Ortiz, Cesar Augusto Medina Tafur, Ligia García, Manuel Oliva, Nilton B. Rojas Briceño

**Affiliations:** ^1^Instituto de Investigación para el Desarrollo Sustentable de Ceja de Selva (INDES-CES), Universidad Nacional Toribio Rodríguez de Mendoza (UNTRM), Chachapoyas 01001, Peru; ^2^Facultad de Ingeniería Civil y Ambiental, Universidad Nacional Toribio Rodríguez de Mendoza de Ama-zonas, Chachapoyas 01001, Peru; ^3^Departamento de Ciencias Biológicas, Universidad Nacional de Trujillo, Trujillo 13007, Peru

## Abstract

The inhabitants of Tunants and Yahuahua face water supply problems in terms of quantity and quality, leading to socio-environmental and health impacts in the areas. The objective of this research, therefore, is to determine the technical and economic feasibility of a proposal for a rainwater harvesting and treatment system for human consumption in the native communities. For the technical feasibility, monthly water demand per family was compared with the amount of water collected in the rainy and dry seasons. In addition, 16 physical, chemical, and microbiological parameters were evaluated at the inlet and outlet of the water system. The economic feasibility was determined by the initial investment and maintenance of the systems; with the benefits, we obtained the net present social value (NPSV), social internal rate of return (SIRR), and cost-effectiveness (CE). Technically, oxygenation and chlorination in the storage tanks allowed for water quality in physical, chemical, and microbiological aspects, according to the D.S. N° 031-2010-SA standard, in all cases. Finally, with an initial investment of S/2,600 and S/70.00 for annual maintenance of the system, it is possible to supply up to six people per family with an average daily consumption of 32.5 L per person. It is suggested that the system be used at scale in the context of native communities in north-eastern Peru.

## 1. Introduction

Safe drinking water and basic sanitation must be available, accessible, safe, acceptable, and affordable for the entire population [1]. The World Health Organisation (WHO) recommends at least 50 L per person per day of water to ensure basic hygiene and nutrition [2]. However, around the world, people die from lack of quality water, especially in rural areas (native and peasant communities) [3]. For instance, Urakusa native community in the Amazonas region (NW Peru) has no basic sanitation services (water supply for drinking and domestic use) and relies on communal silos and latrines for disposal of human waste [4]. In Amazonas, unfortunately, the province of Condorcanqui has the highest percentage of lack of both services (92.3%) [5].

The lack of basic services in rural areas (such as water), together with economic and climatic factors, directly influence chronic child malnutrition and anaemia [6]. The provision of safe drinking water for rural communities must, therefore, be a public priority. However, public projects are unsustainable due to dispersed housing, requiring costly distribution networks [7]. In this situation, rainwater harvesting, storage, and utilisation systems are of paramount importance for those populations that still do not have access to water or have shortages [8].

Thus, rainwater harvesting and rainwater harvesting systems have become an economical and ecological alternative [9]; yet their use has not become widespread due to their long financial return periods [10]. However, there are studies that demonstrate the feasibility of these systems. For example, in Ireland, they focused on treating rainwater to address water depletion due to massive population growth [11, 12]. In Spain, a feasible predictive model was developed for rainwater harvesting in rural communities [13]. In Sydney, average annual water savings are related to annual rainfall and a positive cost/benefit ratio of rainwater storage tanks [14]. In Latin America, because of conditions from northern Chile, Peru, and parts of Ecuador, rainwater harvesting is also feasible [15]. Rainwater storage depends on the size of the tanks and the area, for which technical and economic considerations must be taken into account when choosing the type of storage system [8].

The quality of rainwater must be analysed based on urban areas, physical, chemical, and microbiological factors, which depend on various components suspended in the air [16]. Population growth, forest burning, and industrial expansion cause chemical modification of rainwater [17]. In that sense, rainwater harvesting and treatment is what determines its use, depending on its ability to eliminate enterobacteria, viruses, protozoan cysts, and bacterial spores that can cause disease [18]. Global health depends not only on the quantity of water supplied but also on the water quality; a quarter of the world's population suffers from water-related illnesses [19]. In Urakusa, rainwater quality is poorly prioritised because of the lack of sanitation services [4]. In this sense, rainwater may be used to avoid the use of water from springs and streams, in order to preserve them as they are threatened and highly polluted by human activities [19, 20]. Rainwater treatment has only sense if it is done properly; therefore, the most widely used disinfection method (as part of the treatment) is chlorination due to its easy accessibility and application, as well as its high oxidant capacity expressed in the reduction of organic matter [21].

The cost-effectiveness of rainwater harvesting systems needs to be assessed in order to determine the systems' effectiveness at the user level. The economic analysis allows determining the feasibility of production from rainwater [22, 23]. Water is one of the most important and scarce commodities available to people worldwide, and Peru is no exception in this respect. Many populations are forced to drink from sources whose quality is outside the regulations (D.S. N° 031-2010-SA) leading to health risks for children and adults [24]. In rural Peru, people lack access to safe drinking water; in fact, only 20.0% of the population have access to this service through the public water network [25]. One of the goals of the development objectives (SDGs) is to achieve universal access to safe drinking water, sanitation, and hygiene [26], and Peru is a party to these agreements.

Little have rainwater harvesting projects in native communities been studied, as well as socialisation and prior training for maintenance of the systems implemented [27]. Therefore, it is necessary to implement rainwater harvesting systems in rural areas where access to drinking water is a neglected asset [19]. Based on the above, this research aims, for the first time, to technically and economically validate the rainwater harvesting and treatment system designed for mass use in two native communities (Tunants and Yahuahua) in the Amazonas region (NW Peru).

## 2. Materials and Methods

### 2.1. Study Area and Characterisation of Target Beneficiaries

**Figure 1:**
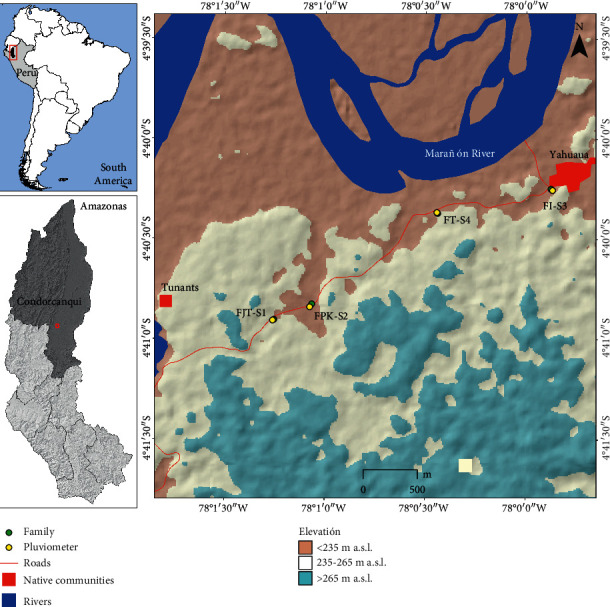
Location map.

The study is located in two native communities, inhabited by Awajún and Wampis peoples (Tunants and Yahuahua), district of Nieva, province of Condorcanqui in the jungle of NW Peru (Figure 1). They are located at an altitude of 196 meters above sea level and an average temperature of 26°C and has an average annual rainfall of 3,121 mm [28]. The communities were created 22 years ago and have a reported population of 217 people in the 2017 census [29].

The province of Condorcanqui faces transportation barriers due to demographic dispersion, as well as it lacks access to basic needs, which include, among others, food, drinking water, and drainage [30]. Their economy is subsistence-based, with land (between 0.5 and 1 ha) dedicated to the cultivation of cassava, bananas, and maize [31]. The characterisation of beneficiaries, on which the systems were designed, was based on interviews aimed at obtaining general data on the population, dwellings, water consumption habits, and evaluating the acceptance level of rainwater harvesting and treatment systems installed in these two native communities.

### 2.2. System Design and Installation

**Figure 2:**
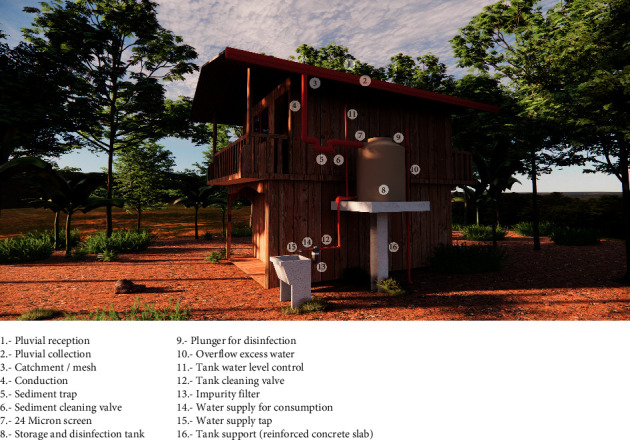
Design of the rainwater harvesting system.

Four stratus rain gauge model 6330 were installed, one in each system (two in each native community). The construction area to set up the systems was determined, ensuring it meets the minimum conditions for the area (place and area of the systems) and the number of users. For the tank construction, three main materials were used: iron, cement, and pipes (PVC). The supporting structure of the tank was built with a mixture of concrete and cement, reinforced with corrugated steel. The design consists of 16 parts indicated in Figure 2, which include a footing of 1 m × 1 m, a central column of section 25 cm × 30 cm and support slab of 1.40 m × 1.40 m, and PVC pipes of 6 and a polypropylene storage tank of 1,100 L with protection against ultraviolet rays (Figure 2). The characteristics of the systems were the same in all four dwellings, except for the size of the column, which was subject to the height of the dwelling. Roof coverings of all dwellings were of galvanised calamine.

### 2.3. Technical Feasibility Determination

To determine the technical feasibility, physical, chemical, and microbiological factors were determined by sampling water, at the inlet and outlet of the systems during three months of the rainy season (December 2019 and January and February 2020) and two months of the dry season (September and October 2020). Sample collection, storage, and transfer, as well as laboratory analysis, were performed according to APHA, AWWA, and WEF [32]. In the rainy season, 264 physicochemical and 72 microbiological samples were analysed, and in the dry season, 64 physicochemical and 32 microbiological samples were analysed. The microbiological parameters were reduced in the dry season, due to the scarce economic resources allocated and the difficult access to the native communities, due to the effects of the COVID-19 pandemic. However, this was not a limitation to continue with the study, given that efforts were made to analyse the TC and CF; the only parameter not taken in the dry season was *Escherichia coli.*

Data collection for pH was in situ, with a Hanna multiparametric water meter model HI 98194, while samples were collected in transparent plastic containers to determine the physicochemical parameters of electrical conductivity (EC), turbidity, total dissolved solids (TDS), total suspended solids (TSS), alkalinity, hardness, nitrates, nitrites, phosphates, sulphates, aluminium, copper, and zinc. Samples were collected for microbiological analysis of total coliforms, faecal coliforms, and *E. coli* in properly sterilised glass bottles with a capacity of 500 ml. They were transported in a cooler with dry ice at a temperature of 5°C. Parameters were analysed at the Water and Soil Laboratory of the Research Institute for Sustainable Development of Ceja de Selva (INDES-CES) of the National University Toribio Rodríguez de Mendoza (UNTRM). Water quality calibration was carried out through chlorination for disinfection at the outlet of the system [33], with commercial bleach in a mechanical way, through the application of a graduated syringe; residual chlorine measurements were carried out with a Hanna HI729 model colorimeter. Likewise, before each sampling, the pH was measured, and the application of potassium hydroxide (KOH) tablets was determined accordingly.

### 2.4. Harvested Water and Projected Catchment Area of the Roof for Water Supply

The volume of rainwater captured in the systems (Vr) was determined by the catchment area of the roof (CR, variable according to the dwelling), the type of roof material (galvanised metal sheet), and its runoff coefficient (Rc, 0.9) [34]. Based on the water harvested, a projection was made of the ideal area to supply water.

(1) $$\begin{array}{c} \text{Vr}=\text{Ce}*\text{Rc}. \end{array}$$

### 2.5. Monthly Water Demand

The monthly water demand per household (Wdh) was assessed. For this, the average amount of water consumption per person (Wcp, 30 L/day [35]), the number of individuals or beneficiaries of the system (Nu), and the period of consumption analysed (Nd, 29, 30, or 31 depending on the month) were identified. The number of individuals per household was obtained through the application of socio-economic surveys [36]. The priorities or activities taken into account were the demand for water at the individual level, including food preparation, personal hygiene, and cleaning of personal items and objects [37].

(2) $$\begin{array}{c} \text{Wdh}=\frac{\left(\text{Nu}*\text{Nd}*\text{Wcp}\right)}{1000}. \end{array}$$

### 2.6. Economic Feasibility Determination

Economic feasibility was determined based on cost-effectiveness, according to geographical aspects (the location of the dwellings and roof area) and costs of water system installation and maintenance. For this, the amount of water supply to dwellings was assessed. The volume of rainwater captured by the roofs (supply) was calculated and then weighed against the members' water needs (demand) [38]. The costs and expenses of the inputs per unit and on average, including the design plans, were taken into account. Inputs and services of the households were also valued.

### 2.7. Economic Viability

To determine the economic viability, a socio-economic evaluation of rainwater harvesting projects was conducted to assess the current situation, current supply, current demand, and problem description [39]. Benefit-cost analysis of the systems installed in the native communities by evaluating the total cost of the system divided into three phases as follows.

### 2.8. Preinvestment and Investment Phase

In the preinvestment phase, the conditioning of the systems and labour costs were taken into account. In the investment phase, the construction of the systems was evaluated, taking into account the components of the catchment area, conduction, storage, filtration, potabilisation, and distribution of rainwater. The opportunity cost of terrain was also considered, as the tank installation requires a large area.

### 2.9. Postinvestment Phase

In this phase, the costs of operation and maintenance were determined, estimating the timescale it should be done.

Cost-benefit: cost-benefit analysis is based on Jianbing's formula [40]:

(3) $$\begin{array}{c} \text{B}/C=\frac{\text{AVB}}{\text{Inv}+\text{PVC}}, \end{array}$$

where AVB is the present value of rainwater benefits, Inv is the investment, and PVC is the present value of costs. The net present social value (NPSV) was carried out to indicate the profitability of the systems, and the projected project horizon was 5 years.

(4) $$\begin{array}{c} \text{NPSV}=\sum_{\left.t=0\right.}^{n}\left.\frac{\text{CFt}}{\left(1+r\right)t}\right., \end{array}$$

where CFt is the year *t* cash flow, *t* is the number of time periods (number of years), *r* means 10% social discount rate, and n is the number of years in assessment horizon minus one. NPSV > 0 indicates that the investment will generate returns. NPSV = 0 indicates that investment project will neither generate profits nor losses. NPSV < 0 indicates that the investment project should be postponed.

### 2.10. Social Internal Rate of Return (SIRR)

It was calculated using the following formula:

(5) $$\begin{array}{c} \text{NPSV}=-I_{0}1+\sum_{t=1}^{n}\frac{Ct}{{\left(1+\text{IRR}\right)}^{t}}=-I_{0}+\;\frac{F_{1}}{\left(1+\text{IRR}\right)}+\cdots +\frac{F_{n}}{{\left(1+\text{IRR}\right)}^{n}}=0, \end{array}$$

where *Ct*: period *t* cash flow, *I*_0_: initial investment (*t* = 0), *n*: number of time periods, and *t*: time period.

### 2.11. Cost-Effectiveness

The cost-effectiveness analysis of a social-economic analysis and nonproject evaluation costs were measured as economic costs, and the results were valued as units of effectiveness [41], assuming that families do not have water, based on the question “How much would a litre of water cost?” and the number of times they carry water, as well as the demand for water per family. A comparison was made between the costs incurred by not having water versus the situation of the satisfaction of having water in the training and treatment systems. The costs were identified in terms of the number of water hauls and the loss of productivity from hauling water (the daily labour cost was taken into account in the internal regulations of the native community of Urakusa). formulas (6) and (7) were used to calculate the daily and annual costs.

### 2.12. Cost-Effectiveness Calculation of Carrying Water from the Stream (Daily)

(6) $$\begin{array}{c} \text{CE}=\frac{Ta}{Jl}*Cj, \end{array}$$

where CE = cost-effectiveness, *Ta* = water carrying time in hours per day, *Jl* = working hours per day, and *Cj* = cost of working time per day.

### 2.13. Cost-Effectiveness Calculation of Carrying Water from the Stream (Annual)

(7) $$\begin{array}{c} \text{Can}=\text{Cad}*\text{Da}, \end{array}$$

where Can = annual cost of water without catchment system, Cad = daily water carrying cost, and Da = number of days per year.

### 2.14. Comparing Projected Costs

We made a comparison for projected costs between the proposed tank water harvesting system (situation with project) versus the tankless water harvesting system, as this is the way the community currently uses the water (situation without project). A 5-year evaluation was carried out, based on the calculation of the annual for each case, including the increase in the number of families (85 families by 2021–2026). The projection (2021–2026) was also calculated using stormwater treatment information and comparing these costs. Additionally, the costs of installing the system with the proposed tank water harvesting system (concrete-based materials) and an installation alternative for families using local materials (materials using native wood) were also described.

We applied a nonparametric Kruskal–Wallis test to identify if there are significant differences between the dry and rainy seasons, using the Minitab 17.1 software (Spanish version).

## 3. Results and Discussion

### 3.1. Characteristics of Beneficiaries

In the selected households of a native community in Amazonas, there are a maximum of 6 family members using water. While for Biswas and Mandal [42], in a remote and rural area of Khulna (Bangladesh), there were a maximum of 4 members, meeting their domestic use throughout the year. Of the selected families, 50% are engaged in agriculture (maize, banana, and cassava cultivation) with an average size of 1 ha per family. They are also engaged in other casual work (day labour) at a daily rate of 40 soles, an amount established by internal rules (apu) within the community.

**Figure 3:**
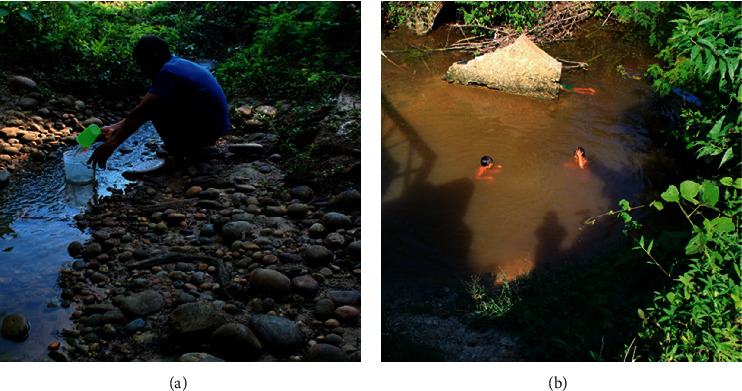
(a) Common water supply features and (b) villagers having their personal hygiene in the stream.

In Tunants and Yahuahua, the inhabitants draw their water from nearby streams or ponds (at an average distance of 75 minutes round journey). Nevertheless, these direct water sources are contaminated by anthropogenic and natural sources [20]. Here, water is commonly carried in gallons and 10 L buckets for daytime supply (Figure 3(a)). However, for their personal hygiene, they usually go directly to the stream (Figure 3(b)). The families also store the water in large containers (between 100 and 1,000 L capacity) to ensure the particles can settle during storage. The water is always boiled before drinking, as the water is contaminated by different types of pollutants, for example, washing powder and faecal dropping from domestic animals. The main reason for nonconstruction of a rainwater harvesting system is the economic factor.

### 3.2. Rainwater Harvesting and Treatment System

#### 3.2.1. Monthly Rainfall

**Figure 4:**
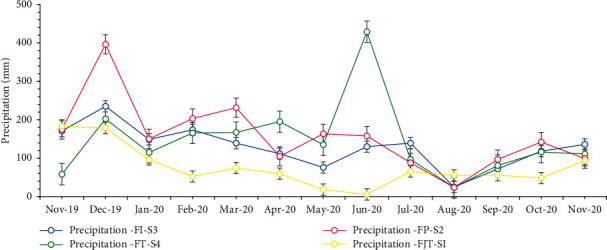
Rainfall evolution: FI-S3 = rain gauge (system three), FT-S4 = rain gauge (system four), FP-S2 = rain gauge (system two), and FJT-SI = rain gauge (system one).

Studies indicate that the annual rainfall in the province of Condorcanqui is between 1,200 and 1,800 mm [27]. The data collected from the rain gauges installed in the study area showed rainfall of up to 396.2 mm in November (Pluviometer-FP-S2) and 429 mm in June (Pluviometer-FT-S4) corresponding to Tunants and Yahuahua, respectively (Figure 4). The lowest rainfall occurred in August (24 mm), corresponding to the Yahuhua area, and 5.76 mm for Tunants. Consequently, rainfall in both communities was consistent and was sufficient capacity for the water catchment systems.

**Figure 5:**
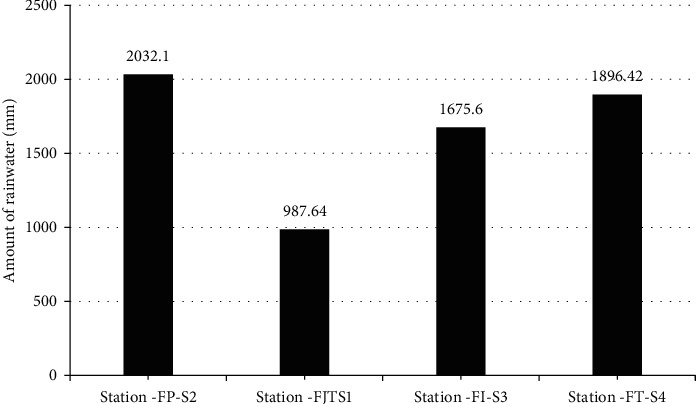
Total annual rainfall reported from rain gauges.

Annual rainfall variations at the stations showed a maximum of 2,032.1 mm and a minimum of 987.64 mm (Figure 5). The National Service of Meteorology and Hydrology of Peru (SENAMHI) shows rainfall values between 1,376.4 and 2,227.8 mm per year at the level of the Nieva district. The values of the installed rain gauges demonstrated the tendencies with respect to the values given by SENAMHI, given the distance of the station from the installed systems.

### 3.3. Technical Feasibility

#### 3.3.1. Amount of Rainwater Collected

The amount of rainwater collected in the systems was not homogeneous (Table 1). In the FPK-S2 family system, there was the highest amount of water collected, with a maximum of 14,263.2 L (December) and a minimum of 311.04 L (June). Rainfall shortage was pronounced in the summer season, during the months of June, July, and August. The amount of rainwater collected is proportional to the area of the roofs. And rainfall is linked to the seasons of the year [15].

### 3.4. Monthly Household Water Demand

Water distribution is unequal; in fact, the poorest areas use about 15 L of water per day and is, of course, influenced by the economic factor [43]. In Mexico, every person has the right to access disposal and sanitation of water equivalent to 30 L per person per day; however, it is still lower than recommended by the World Health Organisation (WHO), suggesting at least 50 L of water per person per day to ensure basic hygiene and nutrition [2]. Household water consumption in the native communities in this research was 71,280 L/year (Table 2) and consumption per person was 32.55 L/day, for an average number of 6 users.

The annual backlog for the FPK-S2 system was 44,244 L. Therefore, it is clear that the water backlog is higher than the demand. The implementation of water recycling systems is proposed, as the water demand is higher than the normative allocation of 30 L per person per day [24]. The backlog in the FI-S3 system was 4,122 L; although the annual backlog is positive, August, September, and October were the most critical period with negative values (−4,455, −2,160, and −257 L, respectively), months in which food preparation is the exclusive priority. In the FJT-SI and FT-S4 systems, the annual lag was −15,258 and −59,473 L. Water deficit was observed in almost all months (Table 2); generally, these negative values are associated with water use in laundry and showering (in months where the lag is negative, water use should be prioritised). For water supply, each month water use should be prioritised, and larger sheds should be installed to capture more water. The 1,100 L storage systems tank was sufficient to supply all of the families' needs for a week, assuming no rain. However, if they only prioritise water for food consumption, it can supply up to 15 days. It was determined that during the rainy months, storage tanks with a maximum capacity of 460 L are needed; therefore, the chosen tanks are the 1,100 L tanks; this is justified because the rains are constant, and there are days when even for the FI-S3 system; only a 15.00 L container is needed to supply water to the family.

### 3.5. Projections of Areas for Rainwater Catchment

The amount of water collected is dependent on the catchment area of the sheds, so roof area measurements have been projected based on water deficit for seasonal low rainfall. Therefore, the average area for installing future investment projects is 89 m^2^ (Table 3). With 89 m^2^ modules, an annual collection of up to 165,884.4 L can be achieved. Unfortunately, the investment in rainwater harvesting may be very costly, making it impossible to install due to economic reasons, thus declining the system's affordability [44]. As such, governments have an obligation to guarantee access to a sufficient quantity of safe drinking water for personal and domestic use [45].

### 3.6. Physicochemical Parameters

The physicochemical parameters (Table 4) for the FPK-S2 and FJT-S1 systems were within the drinking water quality regulations [24] in both periods. In contrast, in the FT-S4 and FI-S3 systems, the parameter aluminium (Al) was the only one that exceeded water quality regulations. The high presence of aluminium may have been influenced by calamine roofs, as well as the combustion of fossil fuels, crude oil, and sources of vehicular traffic close to the installation of the systems [46, 47]. Different pollutants can reach water by wind speed, wind direction, temperature, and the degree of atmospheric stability [48, 49]. In this respect, the quality of rainwater is also influenced by the type of system design [50]. Zinc levels were below the maximum permissible limits (3.0 mg Zn/L) during the rainy season. However, Chubaka et al. [51] found zinc concentrations above 3.0 ppm and copper concentrations above 2.69 ppm. It is possible that this metal is associated with the corrosive action of calamine, such as ultraviolet solar radiation that can damage calamine sheets or structures, causing tiny metal microparticles and paint on the surfaces.

The maximum amount of nitrate (NO_3_) was 3.60 ppm in the FI-S3 system. Nitrate concentration above 50 ppm in water is detrimental to health, and infants may be most affected due to the formation of methemoglobinemia [52].

Rainwater quality varies according to the type of roof and directly influences the parameters of hardness, alkalinity, and turbidity [53]. The maximum turbidity was 1.27 NTU, which is within the Peruvian standard, but it could be due to the number of dry days preceding a rainy event [54]. With respect to total solids, González [54] found parameters between 79 ppm and 94 ppm; for this reason, continuous maintenance of the systems is recommended to reduce the TDS 22.20 mg/L, in the FPK-S2 system. These high and discontinuous values are observed due to the lack of cleanliness of the roof. This dynamic is typical of indigenous communities. TSS varies between 17.60 and 52.83 mg/L. Other studies showed results for total suspended solids ranging from 3 to 304  mg/L. Alkalinity values ranged from 11.13 to 36.57 mg/L CaCO_3_, and all values were very low and acceptable. According to the literature [55, 56], alkalinity is a very important parameter for drinking water, as it buffers rapid pH changes.

The physicochemical results for the low-water season are shown in Table 5, where zinc problems are evident for the FI-S3 and FT-S4 family system, not meeting the standard (D.S. N° 031-2010-SA). However, these heavy metal values in rainwater are lower than values in river water, obtained by the regional government of Amazonas in the community of Kusu Kubaim, in the Nieva district, with high heavy metal values (0.45 and 0.442, respectively) [57]. In the community of Kigkis, in the Nieva district, water from the distribution network showed aluminium (0.527) and iron (0.482) above acceptable limits [57]. Moreover, in the Chiangos community, in the Nieva district, high values of aluminium (0.2062) were found. Aluminium in all systems ranged from minimum and maximum values of 0.2–0.67 mg Al/L, respectively. The problems of heavy metals persist for both periods; technical and economical measures such as oxygenation of the storage tanks should be taken to achieve precipitation of both aluminium and zinc. This is left as a proposal:

- Precipitate aluminium from water. Water is oxygenated in an artisanal way to react with O^−2^ and precipitate as aluminium hydroxide (Al (OH)_3_↓)
- Al^+3^ + O^−2^ ⟶ Al O_3_ + H_2_O ⟶ Al (OH)_3_↓
- Precipitate zinc. It is recommended to oxygenate the water in an artesian way so that it reacts with O_2_ precipitates in the form of zinc hydroxide (Zn (OH)_2_↓)
- Zn^2^ + O^−2^ ⟶ Zn O + H_2_O ⟶ Zn (OH)_2_↓
- Constant cleaning of water storage tanks is also recommended, with at least maintenance every two months. It is an easy method of operation for the users and will bring benefits such as the removal of inorganic (including aluminium that could be present as a precipitate) and organic particles and reduction of turbidity [58].

Table 6 shows the results for microbiological parameters in the rainy season, which were above the water quality regulation (>1,600 NMP/100 mL). In many parts of the world, rainwater does not meet quality standards, and this is attributed to the frequent presence of faecal contamination, mainly from animal origin [59, 60]. High contamination densities are likely to have been caused by the abrupt temperature change during rainfall [61]. Particulates and total coliforms are likely to affect the functioning of the rainwater utilisation system, making ongoing studies a necessity [62].

In low water season, all the results met the standard at the outlet of the system, given that the water samples were taken after treatment (chlorination). The importance of chlorinating the water lies in eliminating microorganisms [63, 64], so disinfection was carried out with commercial bleach at a rate of 5 drops per gallon (of 5 L) and left to stand for 30 minutes before use. When water is not chlorinated, microorganisms may be present in the water [65], as evidenced during the rainy season. With the operation and maintenance of rainwater harvesting systems, the quality of water for human consumption is guaranteed [66]. However, it is recommended that rainwater be chlorinated [67]. Chlorination of stored water reduces the risk of diarrhoea [68]. Therefore, rainwater harvesting systems can improve the quality of life of the inhabitants. In Australia, samples collected from 10 tanks contained *E. coli* in concentrations that exceeded the limit of 150 MPN/100 mL for recreational water quality [69]. Bacteria may be associated with rainfall events and be in connecting pipes, and they can survive and even grow in an open environment, subject to the environmental level of nutrients and conditions such as temperature and pH [70].

pH showed no significant difference (Table 7) and falls within the water quality standards. pH value allows the determination of the degree of contamination caused by sulphur oxides and nitrogen oxides [71]. The pH values obtained are related to the type of storage tank [72], for example, asbestos sheet roofs have pH values of 6.75 [73]. Rainwater pH can vary from weakly acidic (pH 3.1) to weakly alkaline (pH 11.4). In previous studies, the pH of rainwater ranged from 6.6 to 8.26 [74]. In this study, the pH ranged between 6.82 and 7.02.

Rainwater turbidity was below the standard (5 NTU), with average values of 1.24 for the rainy season and 1.58 NTU for the dry season. There were no significant differences between seasons. Turbidity is important to analyse because it influences water clarity, and its presence may be associated with extreme rainfall allowing the presence of suspended solids [16].

Aluminium in rainwater, 0.16 and 0.67 mg/L, exceeded the Peruvian water quality standard for human consumption (0.2 mg Al/L). The statistical analysis shows significant differences between seasons, with higher amounts of aluminium and zinc found during the dry season. The presence of aluminium in water is detrimental to life [16]. The presence of zinc ranged between 2.55 and 3.15, zinc is associated with the type of shed. Acuña [75] found that rainwater collected on galvanised steel roofs is distinguished by higher zinc content (69 to 102 mg/L).

### 3.7. Economic Feasibility

The initial investment of the systems installed is S/2,600 at full cost, and their maintenance is S/70 per year, built of concrete. An alternative rainwater harvesting system is also proposed at a lower cost (S/2,000), with a base constructed of wood, which is abundant in the area, known as Huacapu (*Minquartia guianensis* Aubl). Huacapu is a suitable wood, as it is strong and durable, widely used in construction [76]. The details of the costs in each case (reinforced concrete support and local alternative) are described in Table 8.

The economic evaluation, at a discount rate of 10%, shows an NPSV of S/1,911. The SIRR was above the discount rate, which indicates that future investment in the systems is profitable. The annual benefits to the families are S/1,260, valued at the time spent bringing water to their homes and the cost of consuming clean water (Table 9). In the native community, Juum in the Amazon region, Jiménez [77] evaluated technically and economically a rainwater harvesting system for domestic use and determined that the design of the system is viable and sustainable. The cost of harvested rainwater can be up to nine times lower than desalinated or treated water, and policies are needed to promote the construction and installation of rainwater harvesting systems [78]. Rainwater harvesting is a viable alternative for domestic use and even for irrigation [79]. To reduce costs in treatment systems, it is advisable to place co-layers (grids) that serve as a trap for large particles and leaves from trees that fall on the roof and clog the system [80]. Thus, treated rainwater costs 60% less than drinking water provided by the supplier [79]. The B/C is 1.73 soles, which is cost-effective, but this depends on the project area as it does not agree with the study by Domínguez et al. [79], who found that the cost benefit was $1.34.

### 3.8. Cost-Effectiveness Analysis

The valuation of the cost of water was based on the assumption that how much would the family save to stop carrying water. In this sense, we took into account that the average time spent carrying water is 30 minutes one way and 40 minutes return; the difference is due to the weight of water carried. The average time per family is 2.34 hours/day to carry water just for food preparation and washing dishes (Table 10).

The annual cost of the water supply for food preparation was 4,203 soles, without taking into account the time it takes them in the evenings to go to the streams to have their personal hygiene. Compared to the cost of carrying water in a year, with less than half, they can install a proposed water harvesting and reuse system (S/2,600). As such, access to water has become a management problem to improve the quality of life in rural areas, due to high costs [19]. The lack of water has caused great famines and has led to the mobilisation of entire villages in search of solutions [81]. Native communities are no exception to these social conflicts (access to basic services such as water) [5].

### 3.9. Horizon Assessment for Both without Project and with Project

The 5-year evaluation was carried out on the basis of the total haulage cost per family year (Table 11). The cost of hauling during the evaluation horizon (2021–2026) is shown to be S/2,181.35, which corresponds to the sum of the annual hauling costs in years 0, 1, 2, 3, 4, and 5 (estimated situation without project). It is important to mention that, currently, these costs are covered by the population in the time spent carrying the water. Consequently, they are unable to perform their normal activities (social, family, economic, educational, etc.). Water access, like money, is a fundamental need of any population and is an essential condition for many people to have a better life [82].

Table 12 shows the investment costs over the 5-year evaluation horizon. For the implementation of the rainwater harvesting and treatment system, 89 families were estimated with an annual investment cost of S/2,600.00 per family. The investment in the fifth year would be S/231,400. This evaluation horizon will allow competent bodies to determine the amount of investment in rainwater in order to satisfy the human right to water, without necessarily achieving an economic benefit [82]. In this sense, a cost-effectiveness analysis was useful to value costs that could not be presented in terms of monetary values [83].

Table 13 shows the cost flow over the evaluation horizon, both with and without project. With 10% of the total haulage costs incurred by the inhabitants of the locality (Tunants and Yahuhua), the water supply problem could be solved. This would allow them to cover their needs for human consumption and domestic use water. Socio-economic factors of the population would have positive readjustments, such as human health improvements [83]. Another benefit of rainwater harvesting systems is the reduction of vulnerability to floods and river overflows, which are strategies for the implementation of disaster risk management [39]. Therefore, it is shown that the implementation of rainwater treatment systems projected over 5 years is 262,550 soles. The costs are less than the costs of carrying water from the stream (2,181,357). Future research in the native communities of the Amazon region related to the use of well water is important, as it has shown high potential in other places since it is cheap and quickly accessible in times of drought [84]. Taking into consideration that a limiting factor is microbial contamination of groundwater, which has become a global problem and remains a management challenge as an integrated groundwater model [85, 86].

## 4. Conclusions

Rainwater harvesting for domestic use and human consumption in native communities in Amazonas (NW Peru) is feasible according to technical and economical validation. Rainwater harvesting can supply six family members with daily consumption of 32.5 L per person. Regarding water quality, no significant differences in physicochemical parameters are shown. However, for heavy metals, aluminium showed the most significant difference. A mechanical oxygenation system should be implemented to sediment heavy metals, as it is economical and easy to use. The implementation of rainwater harvesting systems can be an alternative water supply in native communities as it is cheap and accessible. However, water management systems must be implemented for its use, after treatment.

## Figures and Tables

**Table 1:** Monthly quantity of water offered.

Month	Monthly water catchment (L)			
	FPK-S2 (RA = 40 m^2^)	FJT-S1 (RA = 60 m^2^)	FT-S4 (RA = 40 m^2^)	FI-S3 (RA = 50 m^2^)
November 2019	6,296.4	9,877.68	4,834.8	7,672.5
December 2019	14,263.2	9,637.92	7,290	10,575
January 2020	5,414.4	5,214.24	4,140	6,705
February 2020	7,315.2	5,522.58	5,962.32	7,830
March 2020	8,330.4	4,028.4	6,012	6,255
April 2020	3,772.8	3,218.94	7,020	5,031
May 2020	5,882.4	988.2	4,860	3,420
June 2020	5,684.4	311.04	9,612	5,850
July 2020	3,168	3,528.9	3,492	6,255
August 2020	864	2,995.38	864	1,125
September 2020	3,481.2	3,008.88	2,916	3,240
October 2020	5,119.2	2,613.6	4,176	5,323.5
November 2020	3,564	5,076	3,996	6,120
Total	73,155.6	56,021.76	65,175.12	75,402

RA = roof area.

**Table 2:** Monthly water demand per household.

Month	d/m	De *L*/*F*	System (FI-S3)		System (FJT-SI)		System (FPK-S2)		System (FT-S4)	
			*L* (L)	Vol. maximum tank/day (L)	Rez (L)	Vol. maximum tank/day (L)	*L* (L)	Vol. maximum tank/day (L)	*L* (L)	Vol. maximum tank/day (L)
November 2019	30	5,400	2,273	76	4,478	149	896	30	−565	−19
December 2019	31	5,580	4,995	161	4,058	275	−4,684	309	−6,145	37
January 2020	31	5,580	1,125	36	−366	264	4,000	304	−4,435	−10
February 2020	29	5,220	2,610	90	303	292	4,194	397	−5,515	15
March 2020	31	5,580	675	22	−1,552	223	5,929	460	−5,133	28
April 2020	30	5,400	−369	−12	−2,181	158	8,860	421	−4,521	83
May 2020	31	5,580	−2,160	−70	−4,592	5	7,052	417	−3,081	57
June 2020	30	5,400	450	15	−5,089	−165	7,535	441	−3,621	200
July 2020	31	5,580	675	22	−2,051	−226	7,639	349	411	126
August 2020	31	5,580	−4,455	−144	−2,585	−309	5,227	196	−1,677	−26
September 2020	30	5,400	−2,160	−72	−2,391	−399	691	139	−6,213	−110
October 2020	31	5,580	−257	−8	−2,966	−482	−1,408	120	−8,877	−152
November 2020	30	5,400	720	24	−324	−509	−1,688	63	−10,101	−203
Total	396	71,280	4,122	140	−15,258	−722	44,244	3,645	−59,473	27

d/m = Day of month, *L* = lag, De L/*F* = water demand litres/family, and (L) = litres.

**Table 3:** Rainwater harvesting projections of suitable catchment areas.

Dwellings	Dwellings (NP)		Dwellings (P)		Investment/future
	Area of dwellings (m^2^)	Rainwater harvesting (L/year)	Projected area (m^2^)	Rainwater harvesting (L/year)	Recommended area (m^2^)
FPK-S2	40	73,156	70	128,022	89
FJT-S1	60	56,022	85	79,364	
FT-S4	40	65,175	91	148,273	
FI-S3	50	75,402	110	165,884.4	

Nonprojected dwellings (NP) and projected dwelling (P).

**Table 4:** Rainwater quality (mean ± standard deviation) based on monthly evaluation during three months of the rainy season (December 2019 and January and February 2020).

Systems samples (inlet/outlet)	pH (units)	Turbidity (NTU)	TDS (mg/L)	TSS (mg/L)	Alkalinity (mg/L; CaCO_3_)	Hardness (mg/L; CaCO_3_)	Nitrate (mg/L; NO_3_)	Nitrite (mg/L; NO_3_)	Aluminium (mg/L)	Copper mg/L	Zinc mg/L
FPK-S2 (inlet)	6.77 ± 0.08	0.87 ± 0.76	22.20 ± 32.31	43.91 ± 58.10	36.57 ± 24.13	12.81 ± 1.43	1.01 ± 1.12	0.27 ± 0.46	0.07 ± 0.12	0.03 ± 0.04	1.70 ± 2.45
FPK-S2 (outlet)	6.84 ± 0.16	0.53 ± 0.55	12.56 ± 16.41	52.83 ± 68.14	11.13 ± 0.68	13.09 ± 2.76	0.27 ± 0.46	0.17 ± 0.29	0.07 ± 0.11	0.01 ± 0.01	0.65 ± 0.69
FJT-S1 (inlet)	6.80 ± 0.16	1.27 ± 1.78	6.47 ± 4.10	28.83 ± 22.23	14.71 ± 5.89	14.05 ± 4.69	2.25 ± 2.73	0.13 ± 0.23	0.07 ± 0.12	0.02 ± 0.03	2.09 ± 1.99
FJT-S1 (outlet)	6.78 ± 0.15	0.70 ± 0.75	4.20 ± 0.96	17.70 ± 20.36	14.71 ± 5.89	11.16 ± 1.24	3.31 ± 5.73	0	0.08 ± 0.12	0.07 ± 0.01	0.48 ± 0.78
FT-S4 (inlet)	6.63 ± 0.24	0.80 ± 0.72	4.87 ± 1.01	26.60 ± 24.00	22.27 ± 1.36	11.99 ± 1.89	1.75 ± 1.59	0.03 ± 0.06	0.12^*∗*^ ± 0.14	0.01 ± 00	0.84 ± 0.74
FT-S4 (outlet)	6.77 ± 0.16	3.33 ± 4.69	4.40 ± 1.26	21.33 ± 10.07	18.69 ± 6.98	12.81 ± 1.43	0.77 ± 0.75	0	0.07 ± 0.13	0.01 ± 0.01	0.75 ± 0.66
FI-S3 (inlet)	7.09 ± 0.68	0.76 ± 0.80	9.30 ± 5.72	27.67 ± 20.03	18.69 ± 6.98	14.47 ± 5.8	3.60 ± 4.71	0.00 ± 0.01	0.08 ± 0.13	0.01 ± 0.01	2.18 ± 2.55
FI-S3 (outlet)	6.97 ± 0.27	0.65 ± 0.71	4.70 ± 1.73	22.85 ± 18.78	14.71 ± 5.89	10.75 ± 4.35	1.00 ± 0.94	0.00 ± 0.01	0.11^*∗*^ ± 0.20	0.01 ± 0.01	1.03 ± 0.89
D.S. N° 031-2010-SA	6.5–8.5	5 NTU	1,000 mg/L	nc	nc	500 mg CaCO_3_/L	5,000 mg NO_3_/L	3,00 mg NO_2_/L short exposure	0.2 mg Al/L	2 mg Cu/L	3.0 mg Zn/L

^*∗*^Noncompliant; nc = not contemplated in the Peruvian standard.

**Table 5:** Water quality (mean ± standard deviation) based on monthly evaluation during two months of the low water season (September and October 2020).

Systems samples (inlet/outlet)	pH (units)	Turbidity (NTU)	Aluminium (mg/L)	Zinc (mg/L)
FPK-S2 (inlet)	6.95 ± 0.12	1.75 ± 0.21	0.67^*∗*^ ± 0.69	2.51 ± 0.84
FPK-S2 (outlet)	6.99 ± 0.05	1.10 ± 0.42	1.26^*∗*^ ± 1.44	1.82 ± 0.24
FJT-S1 (inlet)	6.80 ± 0.27	1.20 ± 0.28	0.16 ± 0.04	2.06 ± 0.44
FJT-S1 (outlet)	6.81 ± 0.18	1.55 ± 0.21	0.29^*∗*^ ± 0.20	2.59 ± 0.86
FT-S4 (inlet)	7.07 ± 0.13	1.35 ± 0.21	0.21^*∗*^ ± 09	3.12^*∗*^ ± 2.12
FT-S4 (outlet)	7.03 ± 0.12	1.85 ± 0.07	0.24^*∗*^ ± 0.13	3.30^*∗*^ ± 2.31
FI-S3 (inlet)	7.30 ± 0.08	1.90 ± 0.14	0.46^*∗*^ ± 0.28	4.93^*∗*^ ± 3.89
FI-S3 (outlet)	7.29 ± 0.08	2.20 ± 0.28	0.22^*∗*^ ± 0.01	4.92^*∗*^ ± 3.90
D.S. N° 031-2010-SA	6.5–8.5	5 UNT	0.2 mg Al/L	3.0 mg Zn/L

^*∗*^Noncompliant; nc = not contemplated in the standard.

**Table 6:** Microbiological parameter results.

Systems samples (inlet/outlet)	Total coliforms (NMP/100 mL)	Faecal coliforms (NMP/100 mL)	*E. coli* (NMP/100 mL)
*Microbiological parameter (rainy season)*			
FPK-S2 (inlet)	1,600^*∗*^	180^*∗*^	2^*∗*^
FPK-S2 (outlet)	350^*∗*^	130^*∗*^	0
FJT-S1 (inlet)	>1,600^*∗*^	>1,600^*∗*^	17^*∗*^
FJT-S1 (outlet)	13^*∗*^	13^*∗*^	5^*∗*^
FT-S4 (inlet)	920^*∗*^	1,600^*∗*^	4^*∗*^
FT-S4 (outlet)	1,600^*∗*^	<1.8	1,600^*∗*^
FI-S3 (inlet)	1.568^*∗*^	81^*∗*^	>1,600^*∗*^
FI-S3 (outlet)	1.524^*∗*^	23^*∗*^	13^*∗*^

*Microbiological parameter (low water season)*			
FPK-S2 (inlet)	234^*∗*^	99^*∗*^	SN
FPK-S2 (outlet)	<1.8	<1.8	SN
FJT-S1 (inlet)	8.5^*∗*^	7	SN
FJT-S1 (outlet)	<1.8	<1.8	SN
FT-S4 (inlet)	239.5^*∗*^	20.5^*∗*^	SN
FT-S4 (outlet)	<1.8	<1.8	SN
FI-S3 (inlet)	280^*∗*^	84^*∗*^	SN
FI-S3 (outlet)	<1.8	<1.8	SN
D.S. N° 031-2010-SA	<1.8	<1.8	<1.8

^*∗*^Does not comply with the standard (D.S. N° 031-2010-SA); SN = samples not taken.

**Table 7:** Statistical analysis between study periods.

Study periods	pH^ns^ (units)	Turbidity^ns^ (mg/L)	Aluminium^*∗*^ (mg/L)	Zn^ns^ (mg/L)
Rainy season	6.82 + 0.35	1.24 + 0.70	0.13 + 0.10	2.55 + 1.58
Low water season	7.02 + 0.22	1.58 + 0.41	0.37 + 0.34	3.15 + 1.94

ns = no significant difference; ^*∗*^significant difference.

**Table 8:** Installation costs of concrete-based rainwater harvesting systems versus a cheaper wood-based alternative in native areas.

Aspects	Quantity	Unit	Unitary price	Tank support	
				Reinforced concrete support (S/) estimated in total prices	Local alternative (wooden board) estimated in total prices
Cost of land	1	Global		84.00	84.00
Cost of transport	1	Global		1,200.00	600.00

*Cost of installation local labour*					
1 operator from outside the locality^*∗*^	10	day	110	1,100.00	0.00
1 officer from outside the locality^*∗*^	10	day	80	800.00	0.00
1 community labourer^*∗*^	10	day	40	400.00	0.00
1 carpenter from outside the community	13	day	110	0.00	1,430.00
2 community labourers	13	day	80	0.00	1,040.00

*Cost of hardware materials (for concrete base vs. common wood alternative in native areas)*					
1/8-inch thick galvanised metal platen supports	38	Unit	6.8	258.40	258.40
Self-drilling fastening bolts for gutters, hexagonal head	76	Unit	0.6	45.60	45.60
PVC catchment funnel	4	Unit	25	100.00	100.00
PVC pipe, 4 inches	4	Unit	20	80.00	80.00
4 inch × 90 degree PVC elbows	16	Unit	6	96.00	96.00
Codos de PVC 4 × 2 pulgadas, con ventilación	4	Unit	6	24.00	24.00
2-inch PVC plug	4	Unit	2	8.00	8.00
Reduction from 4- to 3-inch PVC	4	Unit	6	24.00	24.00
2 inch × 90 degree PVC elbows	8	Unit	4	32.00	32.00
PVC pipe, 2 inches	8	Unit	13	104.00	104.00
PVC tee, 2 inches	4	Unit	5	20.00	20.00
PVC ventilation cap, 2 inches	4	Unit	5	20.00	20.00
3/4-inch PVC ball valve	4	Unit	15	60.00	60.00
PVC union with 3/4 inch thread	4	Unit	3	12.00	12.00
PVC reduction, 3/4 to 1/2 inch	4	Unit	1.5	6.00	6.00
PVC adapters, 3/4 inch	16	Unit	1.5	24.00	24.00
PVC pipe, 1/2 inch	4	Unit	10	40.00	40.00
3/4-inch PVC pipe	2	Unit	12	24.00	24.00
Mixed PVC joint (thread and spigot), 1/2 inch	4	Unit	2.5	10.00	10.00
1/2-inch PVC spigot union	8	Unit	1.5	12.00	12.00
1/2 inch × 90 degree PVC elbows	10	Unit	2	20.00	20.00
1/2-inch PVC tap	4	Unit	15	60.00	60.00
PVC glue × 1/8 gallon	1	Unit	20	20.00	20.00
Polyethylene tanks with ultraviolet protection, 1,100 L capacity	4	Unit	475	1,900.00	1,900.00
Black annealed wire, 16 gauge	20	kg	5	100.00	0.00
Black annealed wire, 8 gauge	20	kg	5	100.00	0.00
Portland cement type I	32	Bags	26	832.00	0.00
3-inch wood nails	15	kg	8	120.00	0.00
3/8-inch corrugated steel	10	Rod	16	160.00	0.00
1/2-inch corrugated steel	42	Rod	27.5	1,155.00	0.00
1/4-inch corrugated steel	11	Rod	7	77.00	0.00

*Cost of materials in the area*					
River concrete	4.5	m^3^	120	540.00	0.00
Ordinary timber for shuttering	183	p2	4	732.00	0.00
Wood for tank supports	461.5	p2	4	0.00	1,846.00
Total cost (4 systems)				10,400.00	8,000.00
Total cost (1 system)				2,600.00	2,000.00

^*∗*^Category of labour force, existing in civil construction (worker, journeyman, and labourer); p2 = square feet; and Bl = bag of cement weighs 42.5 kilograms.

**Table 9:** Economic analysis of catchment systems.

Benefits and costs	
Initial investment	S/2,600
Systems maintenance (year)	S/70
Benefits (annual)	S/1,260
NPSV	S/1,911
SIRR	36%
B/C	S/1.73

NPSV = net present social value, SIRR = social internal rate of return, and B/C = benefit cost.

**Table 10:** Costs from the social assessment.

Description	Units	Value
Water carrying time	Hours/day	2.34
Daily working day	Hours/day	8
Labour cost	Soles/day	40
Cost per 10 L bucket haulage	Soles/day	11.68
Total haulage cost	Soles/month	350.25
Total haulage cost	Soles/year	4,203.00

**Table 11:** Carrying costs that the locality incurs during the assessment horizon (situation without project).

Year	0	1	2	3	4	5	Total
	2021	2022	2023	2024	2025	2026	2021–2026
Number of families	84	85	86	87	88	89	89
Annual cost (S/)^*∗*^	35,305	35,725	36,145	36,566	36,986	37,406	218,135

^*∗*^Carrying cost without project (S/4,203.00 per family).

**Table 12:** Investment costs over the evaluation horizon (situation with project).

Year	0	1	2	3	4	5	Total
	2021	2022	2023	2024	2025	2026	2021–2026
Number of families	84	85	86	87	88	89	89
Investment cost (S/)^*∗*^	218,400	221,000	223,600	226,200	228,800	231,400	231,400

^*∗*^The investment cost of the systems is S/2,600.00 per family.

**Table 13:** Comparison of costs without project and with project.

Description	Situation without project	Situation with project
Water haulage	2,181,357	0
Investment	0	231,400
OM	0	31,150
Total	2,181,357	262,550

OM: operational and maintenance.

## Data Availability

The data used to support the findings of this study are available from the corresponding author upon request.
